# First reported case of abdominal *Nocardia* pseudocyst in the setting of a ventriculoperitoneal shunt: a case report and review of literature

**DOI:** 10.1186/s13256-025-05555-x

**Published:** 2025-11-06

**Authors:** Jacob Cliett, Matthew Lee, Fernando Vale, Samuel Macomson

**Affiliations:** Department of Neurosurgery, Medical College of Georgia at Augusta University, Augusta, GA USA

**Keywords:** Ventriculoperitoneal (VP) shunt, Pseudocyst, Shunt infection, *Nocardia paucivorans*, Case report

## Abstract

**Introduction:**

Ventriculoperitoneal shunt placement is a common neurosurgical procedure that is used to divert cerebrospinal fluid from the ventricular system to the peritoneal cavity to alleviate hydrocephalus. Ventriculoperitoneal shunts carry the risk of several complications, from mechanical shunt failure and infection to rarer complications such as abdominal pseudocysts. Skin flora has been found to be the cause of most ventriculoperitoneal shunt-related pseudocysts. In this case presentation, we describe what we believe to be the first reported case of *Nocardia* species-induced ventriculoperitoneal shunt-related abdominal pseudocyst.

**Case report:**

A 27-year-old white male with a past medical history of cerebral palsy, congenital seizures, and hydrocephalus treated with four separate ventriculoperitoneal shunts presented to the emergency department for abdominal pain and distension for several months. A computed tomography scan of the abdomen showed a 6.1 cm × 10.7 cm × 14 cm thinly encapsulated fluid collection containing the peritoneal ends of the ventriculoperitoneal shunts, which was indicative of an abdominal pseudocyst. Percutaneous aspiration of the pseudocyst fluid grew *Nocardia paucivorans*. The patient had no history of immunosuppression. The patient underwent externalization of All the shunts along with pseudocyst drainage. Antibiotic therapy was guided by the infectious disease team. Daily cerebrospinal fluid samples were sent until three consecutive cultures demonstrated no growth. The patient later underwent revision of the shunt systems with placement of the distal catheters into the pleural cavities. He was discharged on oral Bactrim and scheduled for a 4-week follow-up in the neurosurgery clinic and the infectious disease clinic. We conducted a systematic review that yielded a total of 27 articles. Our case represents the first case of a *Nocardia*-infected abdominal pseudocyst associated with a ventriculoperitoneal shunt that has been reported in literature.

**Conclusion:**

Abdominal pseudocysts are a rare complication of ventriculoperitoneal shunt placement and can be associated with an underlying shunt infection. We present the first reported case of a *Nocardia*-infected abdominal pseudocyst associated with a ventriculoperitoneal shunt. The patient was successfully treated with shunt revision and antibiotic treatment. This case raises awareness that, in rare cases, uncommon central nervous system pathogens can cause abdominal sequelae in the setting of a ventriculoperitoneal shunt.

## Introduction

Ventriculoperitoneal (VP) shunts are commonly used to divert cerebrospinal fluid (CSF) from the ventricles to the peritoneum for the treatment of hydrocephalus. It is the primary treatment modality for hydrocephalus of varying etiologies, with an estimated 30,000 VP shunt procedures performed yearly in the USA [1, 2]. Although they are relatively common, VP shunts and shunt placement carry the risk of several complications. Mechanical shunt failure, obstruction, and infection are all potential complications that can lead to surgical revision [3].

Infections are a serious complication of VP shunt placement and can lead to revisions, prolonged hospital stays, and increased risk of morbidity and mortality. A single institution retrospective study found a VP shunt-associated infection rate of 5.1% in adult patients and 7.2% in pediatric patients. The most common infectious agents were *Staphylococcus epidermidis* and *Staphylococcus aureus* [4]. Other studies have found a central nervous system (CNS) infection rate in a similar range at 6.1%, with most of the complications occurring in the first year after placement [5]. Abdominal pseudocysts are a rare but potential VP shunt complication. Pseudocysts are loculated intra-abdominal fluid collections that collect at the distal end of the shunt catheter and are frequently associated with infection [6]. The overall incidence of shunt-associated abdominal pseudocysts is estimated to be less than 1% of all cases [7].

We report an unusual case of *Nocardia*-infected abdominal pseudocyst in a patient with a VP shunt, accompanied by a systematic review.

## Methods

### Literature search

An independent reviewer conducted a systematic search of the PubMed database without meta-analysis on 12 November 2024, following Preferred Reporting Items for Systematic Reviews and Meta-analyses (PRISMA) guidelines. The search term “nocardia” as well as “ventriculoperitoneal shunt,” “shunt,” “shunt cyst,” “shunt pseudocyst,” “brain shunt,” “abdomen cyst,” “abdomen shunt,” and “abdomen pseudocyst” were used to screen the database. There were no filters, limits, or temporal restrictions. We excluded nonclinical studies, cadaveric studies, nonhuman studies, editorials, technical notes, articles not in English, conference proceedings, articles describing *Nocardia*-infected pseudocysts in the absence of a VP shunt, and articles describing *Nocardia* brain abscesses with no shunt involvement. The reviewer conducted a manual screen of titles and abstracts on articles describing abdominal *Nocardia*-infected pseudocysts in the setting of a VP shunt. Articles that did not meet exclusion criteria were then selected for a full-text review. The reviewer conducted a qualitative search of Google Scholar and a review of included articles’ bibliographies.

## Results

### Case presentation

A 27-year-old white male presented to the emergency department for abdominal distension and lower back pain. He Also reported constipation, decreased appetite, and concentrated urine. He denied fevers, chills, severe headaches, or urinary incontinence. His past medical history was significant for cerebral palsy and congenital seizures. His past surgical history included right occipital lobectomy for seizure disorder and VP shunt placement for hydrocephalus management. He required approximately 25 revisions of the VP shunt. One of the shunt revisions was related to an infection secondary to *Staphylococcus aureus* with no associated pseudocyst. His last VP shunt revision surgery was 10 years prior at an outside hospital. The patient was most recently evaluated in the neurosurgery clinic 5 years prior to presentation with no complaints related to the VP shunt. On physical examination, the patient was afebrile with normal heart rate, blood pressure, and oxygen saturation. The patient was awake and Alert with no focal neurologic deficits. There was no erythema or tenderness at any shunt site. His abdomen was tender to palpation diffusely without rebound tenderness. Initial white blood cell count was 8.2/mm^3^ with a segmented neutrophil differential of 82%. All other laboratory values were normal.

**Fig. 1:**
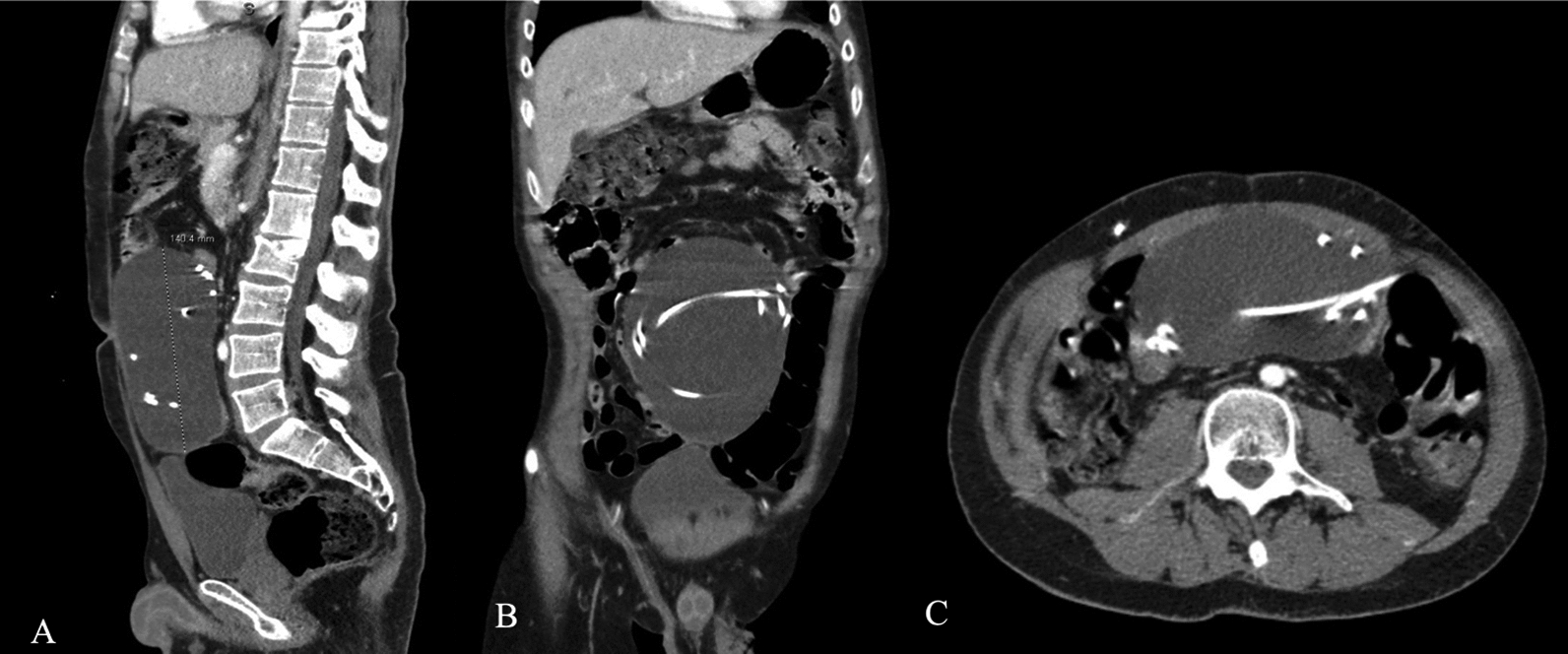
**A** shows a sagittal view of the abdominal pseudocyst measuring 140.4 mm in length; **B** shows a coronal view; **C** shows an axial view

A radiological workup consisting of skull and chest X-rays along with computed tomography (CT) scans of the head and abdomen were obtained. An abdominal CT scan (Fig. 1) showed a 6.1 cm × 10.7 cm × 14 cm thinly encapsulated fluid collection containing the peritoneal end of the VP shunt, which was consistent with an abdominal pseudocyst. Head CT showed a mild increase in ventricular size consistent with hydrocephalus. Neurosurgery was consulted and recommended percutaneous image-guided drainage and aspiration of the pseudocyst. The pseudocyst fluid was cultured and subsequently grew *Nocardia paucivorans* after 5 days. The patient underwent externalization of all VP shunts and laparotomy for pseudocyst evacuation and debridement. The infectious disease service was consulted and recommended Bactrim DS 224 mg intravenously twice daily for 1 week followed by Bactrim DS two tablets by mouth twice daily for 6 weeks. Daily CSF samples were sent for culture until three consecutive cultures showed no growth. After completion of the initial antibiotic course and three consecutive negative culture growths, the patient underwent replacement and internalization of the right frontal and right parietal distal catheters into the pleural cavity. The right frontal ventricular catheter was replaced, while the parietal ventricular catheter was not replaced owing to adherence to the brain and increased risk of intracranial hemorrhage. The patient underwent left-sided distal shunt revision and internalization into the pleural cavity 2 days later for his left frontal and occipital shunts. The left occipital ventricular catheter was also replaced.

The patient recovered well from all procedures. The patient was discharged on postoperative day 2 from shunt replacement. His entire hospital stay was 2 weeks. He was discharged on oral Bactrim per infectious disease recommendations. At outpatient follow-up 4 weeks later, he was doing well, and his surgical sites were well healed.

### Literature review

**Fig. 2:**
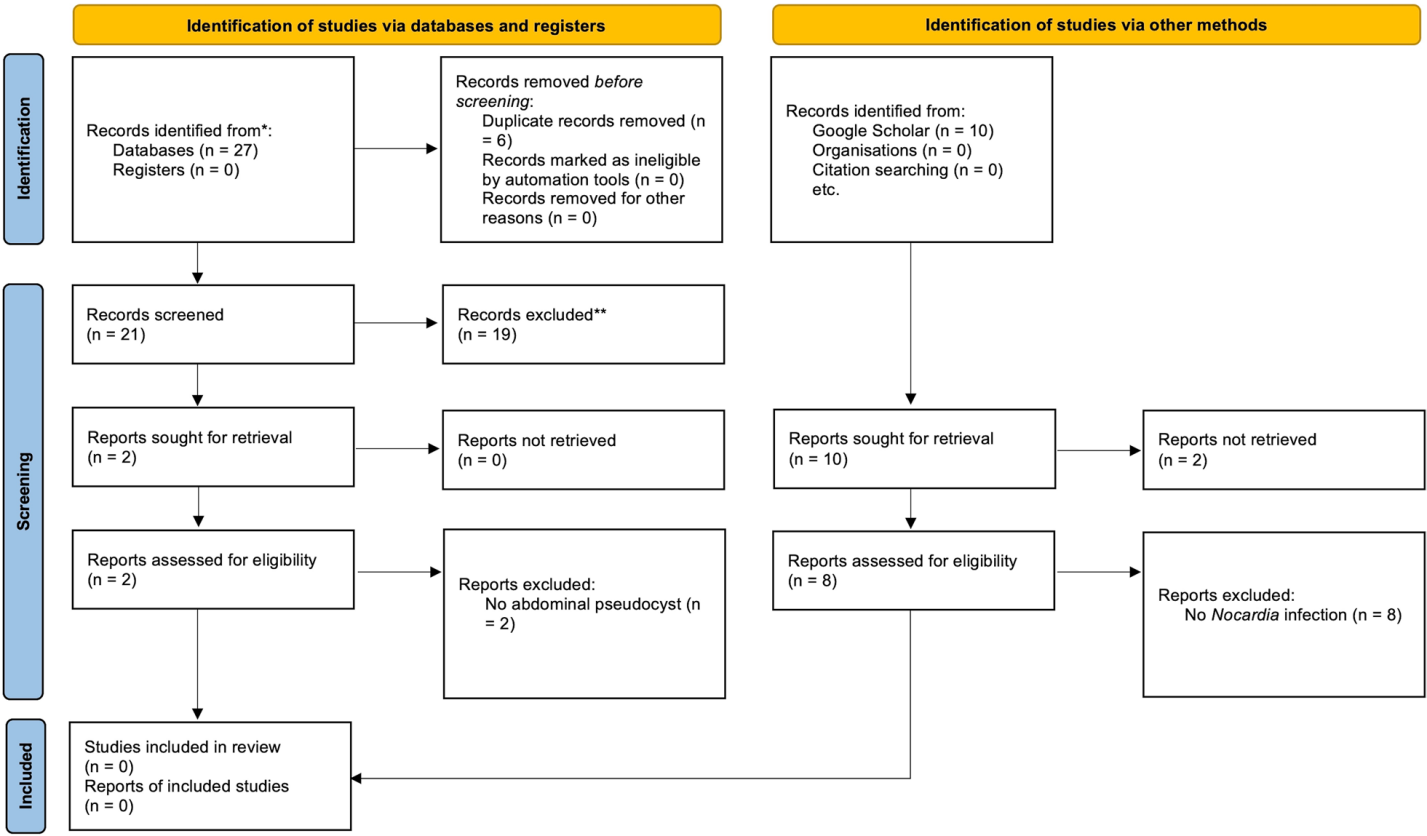
Flowchart of literature review

The initial literature search of the PubMed database yielded a total of 27 publications. The PRISMA flowchart diagram of our search is shown in Fig. 2. After removing duplicates and an initial screening of titles and abstracts, two articles were selected for a full-text review. Of these full-text reviews, neither of the articles were found to meet inclusion criteria. A supplemental search of Google Scholar yielded no additional articles that met the inclusion criteria. Our case represents the first-ever reported *Nocardia*-infected abdominal pseudocyst associated with a VP shunt.

## Discussion

### *Nocardia* CNS infections

*Nocardia* species are weakly acid-fast aerobic Gram-positive bacteria that are commonly found in soil. They can cause opportunistic infections in immunocompromised individuals and commonly affect the lungs and cutaneous tissues through granulomatous inflammation. Although most patients with nocardiosis are immunocompromised, prior studies have shown that approximately one third of affected patients are immunocompetent individuals [8]. Each year, there are approximately 500–1000 cases of nocardiosis in the USA [9]. Disseminated nocardiosis can affect the CNS, and previous studies have shown that 15–44% of patients with systemic nocardiosis have cerebral abscesses owing to *Nocardia* species [10]. Cerebral abscesses are relatively uncommon, with an estimated worldwide incidence of 0.3–1.3 per 100,000 persons per year [11]. *Nocardia* species are estimated to account for only 2% of all cerebral abscesses [12]. Prior case reports have disclosed other CNS presentations, including meningitis, vasculitis, leptomeningeal enhancement, and nocardial infections of the spine [13]. *Nocardia* species are capable of invading and surviving in a variety of host cells, including epithelial cells and macrophages. Several species have been shown to cause microglial-mediated neuroinflammation and can enter the brain parenchyma by crossing capillary endothelial cells [14]. Notably, *Nocardia* species have been implicated in catheter-associated infections and bacteremia, including central venous catheters [15]. Abdominal nocardiosis and abscesses have also been reported in literature, both as an isolated finding and in association with a peritoneal dialysis catheter [16]. No reported findings of abdominal nocardiosis have been found in association with a VP shunt such as in our patient.

### Etiology of shunt-associated abdominal pseudocysts

The exact pathophysiology behind abdominal CSF pseudocysts is not clear, but a common proposed mechanism in literature is an inflammatory reaction that leads to encapsulated fluid collection in the peritoneal cavity. Some potential causes could be prolonged peritoneal inflammation due to catheter placement, immune-mediated catheter rejection, an extensive shunt revision history, and current or prior shunt-related infection [17]. The infection rate for abdominal pseudocysts is approximately 42%, and infection is believed to be the main cause of their formation. Commonly implicated bacteria include *Staphylococcus epidermidis* and *S. aureus*, as well as commensal skin organisms such as *Cutibacterium acnes* [18]. In the case of a sterile CSF culture, several authors have postulated that the presence of a pseudocyst should be treated as a shunt infection, as the infection could be transient or latent on presentation [19].

### Present case

We presented a case of VP shunt-associated *Nocardia*-infected abdominal pseudocyst. Our patient presented to the emergency room (ER) with abdominal complaints, but, notably, there were no obvious signs of increased intracranial pressure suggesting shunt malfunction. Many reported cases of shunt-associated abdominal pseudocysts in adult patients describe an initial presentation of mainly abdominal complaints rather than hydrocephalus symptoms [20]. Clinicians should have a high index of suspicion for a shunt complication in patients with VP shunts that present with abdominal complaints. Early imaging evaluation with ultrasonography or a CT scan can confirm the diagnosis and direct further management [7, 21].

In 2005, Mobley III *et al*. proposed a treatment algorithm for abdominal pseudocysts. In their proposed algorithm, treatment decisions are guided by a preliminary shunt tap to evaluate for infection. If an infection is confirmed, the entire shunt is removed. A sterile ventricular drain is placed, and the patient is treated with appropriate antibiotics until cultures are negative for three consecutive days. If infection is initially unclear based on the CSF tap, the distal shunt catheter is externalized while the pseudocyst is cultured for further infectious workup. After the infection is appropriately treated, or ruled out, the VP shunt is replaced. If needed, a different distal catheter destination is used (such as ventriculopleural shunt) depending on whether the abdomen is suitable for replacement [17]. Abdominal pseudocysts that were treated with peritoneal shunt replacement had a high rate of recurrence, ranging from 27–62%. The fibrous scarring and prolonged inflammation associated with the formation of an abdominal pseudocyst may indicate that the peritoneum is not equipped to appropriately reabsorb CSF [17, 22]. Our patient’s treatment course was very similar to the algorithm proposed by Mobley III *et al*. and used CSF cultures and pseudocyst cultures to determine whether there were infections throughout the shunt system. The patient’s extensive shunt revision history, as well as the size and underlying *Nocardia* infection in the pseudocyst, was what drove the decision to excise the pseudocyst and place ventriculopleural shunts instead of repositioning new ventriculoperitoneal shunts. A definitive source of the *Nocardia* infection in our patient was never identified.

## Conclusion

Abdominal pseudocysts are a rare complication of VP shunts. They are associated with multiple shunt revisions, prolonged peritoneal inflammation, and underlying infection. We present the first reported case of a *Nocardia*-infected abdominal pseudocyst associated with a VP shunt. The patient was successfully treated with antibiotic therapy and shunt externalization followed by replacement/revision of the shunt systems into the pleural cavities. Our case suggests that uncommon CNS pathogens have the potential to cause abdominal complications in the setting of a VP shunt. In patients with a VP shunt and abdominal symptoms, clinicians should have a low index of suspicion for initiating an infectious workup.

## Data Availability

Data sharing is not applicable to this article as no datasets were generated or analyzed during the current study.
